# Low-Temperature Ethanol Gas Sensor Based on MoO_3_/Nb_2_C MXene Composite via Crystal Engineering and Facet Release

**DOI:** 10.3390/s26113450

**Published:** 2026-05-29

**Authors:** Baohui Zhang, Haoyu Zhou, Xiaowu Zhu, Haoxiang Chen, Yang Yang

**Affiliations:** 1 School of Integrated Circuits, Dongguan University of Technology, Dongguan 523808, China; zhangbaohui@dgut.edu.cn (B.Z.); 241219448@dgut.edu.cn (H.Z.); 231219019@dgut.edu.cn (X.Z.); 2 State Key Laboratory of Electronic Thin Films and Integrated Devices, University of Electronic Science and Technology of China, Chengdu 611731, China

**Keywords:** MoO_3_, Nb_2_C MXene, gas sensor, ethanol, facet engineering, heterojunction

## Abstract

High-performance ethanol sensors with low power consumption show critical applications in environmental monitoring, personal health diagnosis, industry and traffic safety. Herein, MoO_3_/Nb_2_C MXene heterojunction gas-sensing materials were constructed via a one-step hydrothermal method for MoO_3_ nanotube synthesis. The dominant facets of MoO_3_ were shifted from the (040) orientation in MoO_3_ nanotubes to the (110) and (021) orientations in the MoO_3_/Nb_2_C MXene composite. Nb_2_C nanosheets provide a large number of crystallization sites, preventing the growth of MoO_3_ nanotubes during synthesis, inducing a strategic facet release. The sensing performance shows MoO_3_/Nb_2_C MXene composite reduces the operating temperature down to 120 °C. The 15 wt% Nb_2_C MXene-precursor-mixed MoO_3_ sensor exhibits an enhanced response of 6.1 toward 100 ppm ethanol, which is higher than that of pristine MoO_3_ nanotubes at 120 °C, with response and recovery times of 19 s and 72 s, respectively. The sensors show high selectivity toward ethanol over other VOC gases and good long-term stability over 30 days. This work confirms that crystal engineering is an effective method for reducing operating temperature and enhancing gas-sensing performance, and the sensor shows potential application for ethanol sensing.

## 1. Introduction

Ethanol (C_2_H_5_OH) is widely used as a fundamental feedstock in chemical synthesis, food processing, and pharmaceutical industries but is also extensively employed as a solvent, fuel additive, and disinfectant [1]. Due to its high volatility and flammability, ethanol vapor readily forms explosive mixtures with air, particularly in high-temperature industrial environments. Once ignited, this can lead to catastrophic fire or explosion accidents, posing a severe threat to both industrial infrastructure and human safety [2]. Furthermore, prolonged exposure to ethanol vapor at concentrations as low as 25 ppm may induce respiratory irritation, visual impairment, and severe damage to the central nervous system [3]. Additionally, ethanol detection represents a pivotal technological approach in traffic enforcement for preventing driving under the influence (DUI) and reducing traffic accident rates [4]. Consequently, the development of highly sensitive and low operating temperature ethanol gas sensors has emerged as an urgent necessity in the fields of environmental monitoring, public health and safety protection.

Metal oxide semiconductor (MOS) gas sensors, such as WO_3_ [5], SnO_2_ [6], TiO_2_ [7], ZnO [8], Co_3_O_4_ [9], and In_2_O_3_ [10], have garnered significant attention due to their superior sensitivity, rapid response, facile fabrication processes, and cost-effectiveness [11]. Among various MOS materials, molybdenum trioxide (MoO_3_) is regarded as an ideal candidate for constructing high-performance gas-sensing devices, owing to its unique layered structure, rich chemical valence states, excellent thermal stability, and suitable band gap [12]. However, most pristine MoO_3_ gas sensors typically require elevated operating temperatures (300–400 °C) to activate surface reactions [13]. This requirement not only results in high power consumption and a shortened device lifespan but also poses severe safety hazards during the detection of flammable and explosive gases. Recent research efforts have been concentrated on improving the performance of MoO_3_-based sensing materials, particularly by reducing the operating temperature and improving sensitivity. This has been achieved through the control of morphology and reduction in dimension as evidenced by studies that have explored various preparation methods and the resulting performance enhancements. For instance, Li et al. synthesized ultrathin MoO_3_ nanobelts with a thickness of only 10 nm via a sulfur-induction strategy. This morphology significantly increased the specific surface area, resulting in a three-fold enhancement in response to 500 ppm ethanol at 350 °C [14]. Similarly, Li et al. introduced an in situ growth strategy to directly synthesize hierarchical α-MoO_3_ flower-like structures on alumina ceramic tubes. Composed of assembled nanosheets, these structures demonstrated an ultra-low detection limit of 1 ppb for triethylamine at a low temperature of 133 °C [15]. In the same year, Tian et al. prepared La-doped 3D MoO_3_ microspheres using a chitosan-assisted hydrothermal method. The sensor based on 1 wt% La-doped MoO_3_ exhibited the highest gas-sensing response to triethylamine (TEA); at 240 °C, the response to 20 ppm TEA was approximately 30, with response and recovery times shortened to 10 s and 29 s, respectively [16].

Reducing dimensions and controlling morphology represent effective strategies for lowering the operating temperature of MoO_3_-based gas sensors. However, constrained by the intrinsic wide band gap of MoO_3_, its carrier concentration at room temperature is extremely low, resulting in excessive device resistance and restricted electron injection efficiency [17]. Consequently, constructing highly conductive heterojunction networks to facilitate carrier transport is of paramount importance. For instance, Chang et al. synthesized h-MoO_3_ nanorods on graphene films via a chemical bath deposition method. Benefiting from the synergistic effect between h-MoO_3_ and graphene, the composite sensor exhibited a 40% increase in conductivity and achieved high sensitivity for NH_3_ detection at room temperature with a rapid response time of 46.8 s [18]. Similarly, Zhang et al. prepared MoO_3_/Ti_3_C_2_T_x_ MXene nanocomposites using a simple hydrothermal synthesis method. Leveraging the doubled specific surface area and the Schottky contact at the heterojunction interface, their device realized the effective monitoring of ethanol at a low temperature of 100 °C, with a response/recovery time of 10 s/49 s [19]. Furthermore, Ma et al. constructed a p-n heterojunction based on electrospun MoO_3_ nanofibers and 2D Ti_3_C_2_T_x_ MXene nanosheets. Due to the high electron transfer efficiency of the 1D/2D composite structure and the establishment of the p-n interface, the sensor demonstrated excellent room-temperature response to trimethylamine (TMA) with an ultra-fast response time of only 10 s [20]. Additionally, Yan et al. fabricated MoS_2_/MoO_3_ surface heterostructures through the surface oxidation of MoS_2_ nanosheets using an aqueous solution. This flower-like heterojunction fully utilized the modulation of the p-n junction and the unique 3D morphology to achieve high-performance NO_2_ sensing at room temperature, exhibiting a response of 18.9% to 1 ppm NO_2_ [21].

Herein, MoO_3_/Nb_2_C MXene nanocomposites were synthesized via a solvothermal method with varying mass ratios to improve performance. Transmission electron microscopy (TEM) reveals that the incorporation of Nb_2_C MXene induces a morphological evolution of MoO_3_ from one-dimensional (1D) nanorods to defect-rich cluster-like nanostructures. X-ray diffraction (XRD) confirms that the dominant crystal facets of MoO_3_ transition from the thermodynamically stable (040) basal plane to the (110) and (021) edge facets. The resistive gas sensors were fabricated and tested for gas-sensing performance. The MoO_3_/Nb_2_C MXene composite devices reduce operating temperature. Furthermore, the potential ethanol adsorption and electronic sensing mechanism of MoO_3_/Nb_2_C MXene composites are discussed in detail.

## 2. Materials and Methods

### 2.1. Materials and Synthesis

All the reagents were utilized as supplied without additional purification treatment. The Nb_2_AlC powder was purchased from Jilin 11th Technology Co., Ltd. (Jilin, China). Sodium molybdate dihydrate (Na_2_MoO_4_·2H_2_O) and sodium chloride (NaCl) were bought from Shanghai Aladdin Biochemical Technology Co., Ltd. (Shanghai, China). Hydrochloric acid (HCl), hydrofluoric acid (HF, 40 wt%), and ethanol were obtained from Sinopharm Chemical Reagent Co., Ltd. (Shanghai, China). The resistivity of the DI water employed throughout the whole experiment was around 18 MΩ·cm.

The Nb_2_C MXene nanosheets were prepared by selectively etching Al from the Nb_2_AlC MAX phase [22]. Specifically, 2 g of Nb_2_AlC powder was slowly added to 20 mL of 40 wt% HF solution and stirred at 60 °C for 48 h. The resulting product was repeatedly washed with DI water via centrifugation (8500 rpm, 6 min) until the pH reached 7.0, and then dried in a vacuum oven at 60 °C for 8 h.

**Figure 1:**
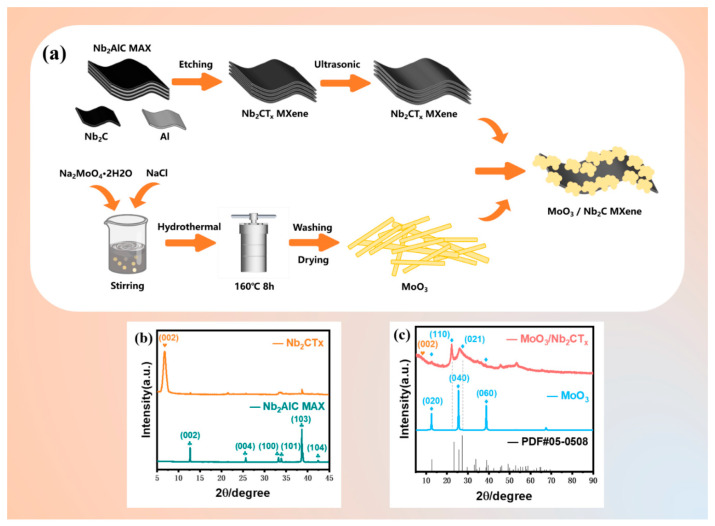
(**a**) Schematic illustration of the synthesis process for MoO_3_/Nb_2_C MXene composites; XRD patterns of (**b**) Nb_2_AlC MAX and Nb_2_C MXene; (**c**) pure MoO_3_ and MoO_3_/Nb_2_C composite.

The MoO_3_/Nb_2_C nanocomposites were synthesized via a facile hydrothermal method, as schematically illustrated in Figure 1a. To investigate the effect of MXene content on the sensing performance, a series of composites with different mass ratios was prepared. First, calculated amounts of the as-prepared Nb_2_C MXene powder were ultrasonically dispersed in 22 mL of DI water. Then, 90 mg of Na_2_MoO_4_·2H_2_O and 40 mg of NaCl were dissolved in the aforementioned suspension. Following 20 min of continuous stirring, the pH of the mixture was precisely adjusted to 1 by adding a 6 M HCl solution. Subsequently, the precursor solution was transferred into a 50 mL Teflon-lined stainless-steel autoclave and maintained at 150 °C for 10 h. The resulting precipitates were collected by centrifugation, washed with DI water six times, and dried at 45 °C for 18 h. The final products were labeled as MX-5, MX-10, MX-15, and MX-20, corresponding to Nb_2_C mass percentages of 5 wt%, 10 wt%, 15 wt%, and 20 wt%, respectively.

### 2.2. Material Characterization

The crystal structures of the as-prepared samples were analyzed by X-ray diffraction (XRD; Rigaku D/Max 2550, Akishima, Japan) using Cu Kα radiation (λ = 1.5418 Å) in the 2θ range of 5–80°. The surface morphology and microstructure were observed via scanning electron microscopy (SEM; Sigma 300 Zeiss, Oberkochen, Germany) with an acceleration voltage of 15 kV. Elemental composition and distribution were determined using energy-dispersive X-ray spectroscopy (EDS) coupled with the SEM system. Transmission electron microscopy (TEM; FEI Tecnai G2 F20 S-Twin, Hillsboro, OR, USA) was conducted at an operating voltage of 200 kV. The surface chemical states were investigated by X-ray photoelectron spectroscopy (XPS) on a Thermo Fisher Scientific K-Alpha spectrometer (Waltham, MA, USA) with an Al excitation source; for XPS measurements, the samples were prepared by drop-casting the dispersion onto silicon substrates.

### 2.3. Gas-Sensing Performance Testing

The gas sensors were fabricated by drop-coating the active materials onto alumina substrates pre-equipped with silver interdigitated electrodes (13.4 × 7.0 × 0.635 mm). Before coating, the substrates were ultrasonically cleaned in ethanol (0.789 g/mL, 99.7% from Sinopharm) and DI water. The materials were dispersed in DI water and ultrasonicated for 10 min to form a uniform slurry. After coating, the sensing films were dried at 80 °C for 2 h and subsequently annealed at 250 °C for 30 min to ensure structural stability. The film was about 50 μm after annealing.

**Figure 2:**
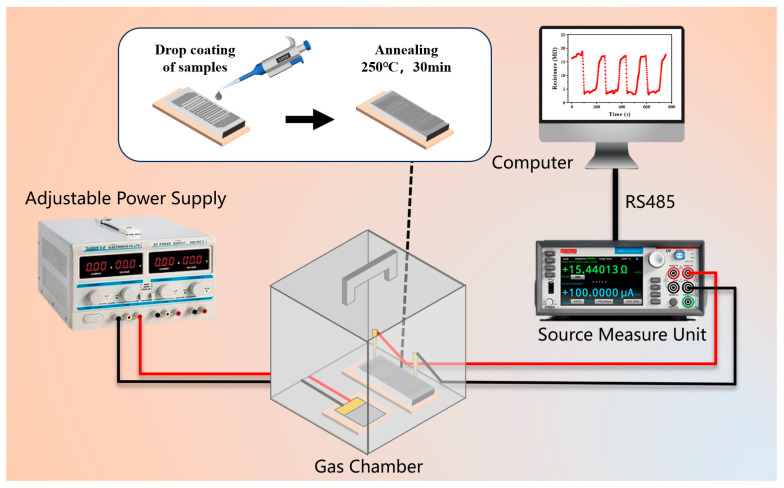
Schematic diagram of the experimental gas-sensing measurement system.

Gas-sensing performance was evaluated in an 18 L acrylic chamber using air as the reference gas, and a schematic diagram of the experimental gas-sensing measurement system is shown in Figure 2. The sensor resistance was monitored using a Keithley 2400 SourceMeter (Cleveland, OH, USA). Target gas concentrations (*C*) were controlled by evaporating calculated volumes of liquid (*V_x_*) according to the formula:

(1) $$V_{x}=\frac{V\cdot C\cdot M}{24.2\cdot d\cdot \rho }$$

In this equation, *V* is the volume of the chamber (18 L). *M* is the molar mass of ethanol, which is 46. *ρ* is the liquid density and *d* means liquid purity (here is absolute ethanol).

The sensor response toward ethanol was defined as S = R_a_/R_g_, where R_a_ and R_g_ represent the baseline resistance in air and the resistance in the target gas, respectively [23]. All sensing measurements were repeated at least three times to ensure reproducibility.

## 3. Results

### 3.1. Materials Characterization

The crystal structures of the as-prepared α-MoO_3_, Nb_2_C MXene, and MoO_3_/Nb_2_C composites were characterized via XRD, as shown in Figure 1. The XRD pattern of pure α-MoO_3_ displays intense, sharp diffraction peaks at 12.8°, 25.7°, and 39.0°, which are indexed to the (020), (040), and (060) planes of the orthorhombic phase (JCPDS No. 05-0508). The sharp intensity of the (0k0) peaks suggests a preferred orientation along the ~13° to 7.2° after HF etching, accompanied by the disappearance of the Al-related (104) peak, confirming the successful removal of Al layers and the expansion of interlayer spacing due to surface terminations (-O, -F, and -OH) [24].

A thermodynamically stable direction occurred during synthesis, constructing one-dimensional single-crystalline nanorod morphology. For the Nb_2_C MXene, the (002) reflection shifted.

Regarding the MoO_3_/Nb_2_C composites (Figure 1c), while the α-MoO_3_ framework is preserved, a significant redistribution of peak intensities is observed. Specifically, the relative intensity of the (040) peak has almost vanished, while the signals from the (110) and (021) facets are significantly enhanced. Compared to the sharp (040) peak corresponding to the size-similar single-crystalline α-MoO_3_ nanorod, the broadening (110) and (021) facets demonstrate the hydrothermal polycrystalline growth process with distinctly varying sizes.

**Figure 3:**
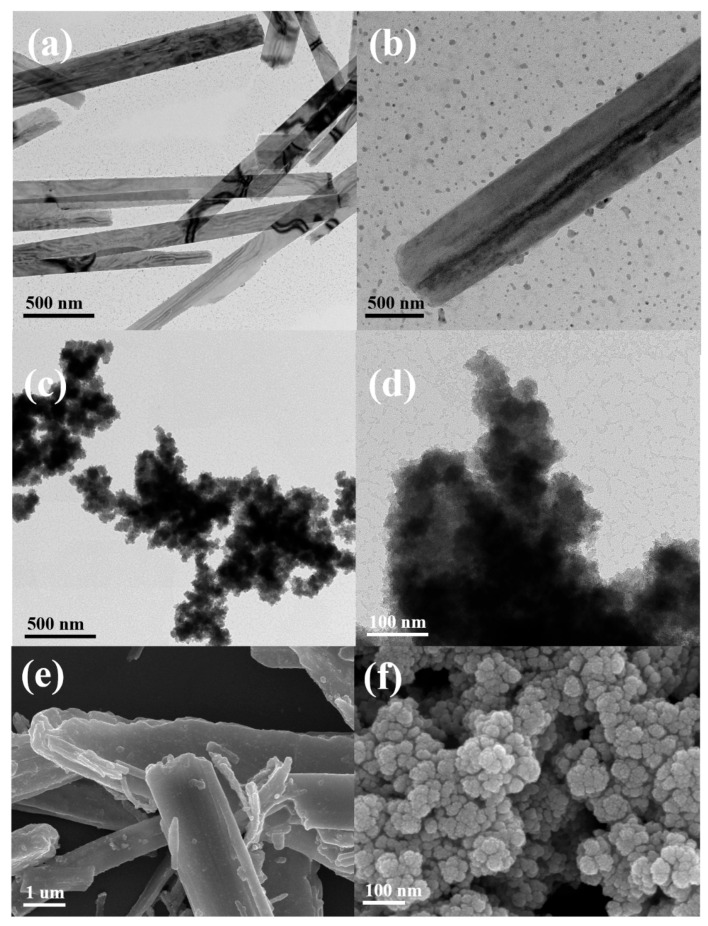
TEM images of (**a**,**b**) pure α-MoO_3_ nanorods and (**c**,**d**) MoO_3_/Nb_2_C MXene nanoclusters. SEM images of (**e**) pure α-MoO_3_ nanorods and (**f**) MoO_3_/Nb_2_C MXene nanoclusters.

The surface morphology and microscopic structure of the samples were characterized by SEM and TEM. Figure 3e illustrates the SEM image of pristine MoO_3_, exhibiting a typical one-dimensional nanorod morphology with a smooth surface and high aspect ratio. Further structural details are revealed by TEM in Figure 3a,b, confirming the presence of a uniform rod-like structure with diameters around 150 nm. The observed XRD patterns align well with these findings, as the pronounced (020), (040), and (060) diffraction peaks indicate a preferential orientation of growth along the [0k0] direction. This orientation results in the prevalence of chemically inert (040) facets in the nanorod structure [25]. Upon compounding with Nb_2_C MXene, a distinct morphological transformation is observed. As shown in the SEM image (Figure 3f), the original long nanorods disappear, and instead, numerous MoO_3_ nanoclusters are densely and uniformly distributed on the surface of the wrinkled Nb_2_C nanosheets. This significant change is further corroborated by the TEM images in Figure 3c,d, where the layered structure of Nb_2_C serves as a robust supporting substrate, effectively anchoring the MoO_3_ nanostructures.

The transition from 1D nanorods to 0D nanoclusters indicates that the introduction of Nb_2_C MXene acts as a “structural template” that restricts the anisotropic growth of MoO_3_. This morphological evolution provides supportive evidence for the facet reconfiguration inferred from the XRD analysis; the suppression of the [0k0] growth direction leads to the shrinkage of (040) facets and the increased exposure of highly active (110) and (021) facets. Such a structural reconfiguration is expected to increase the specific surface area and expose more unsaturated active sites, which are beneficial for the adsorption and surface reaction of ethanol molecules, thereby potentially enhancing the gas-sensing performance.

**Figure 4:**
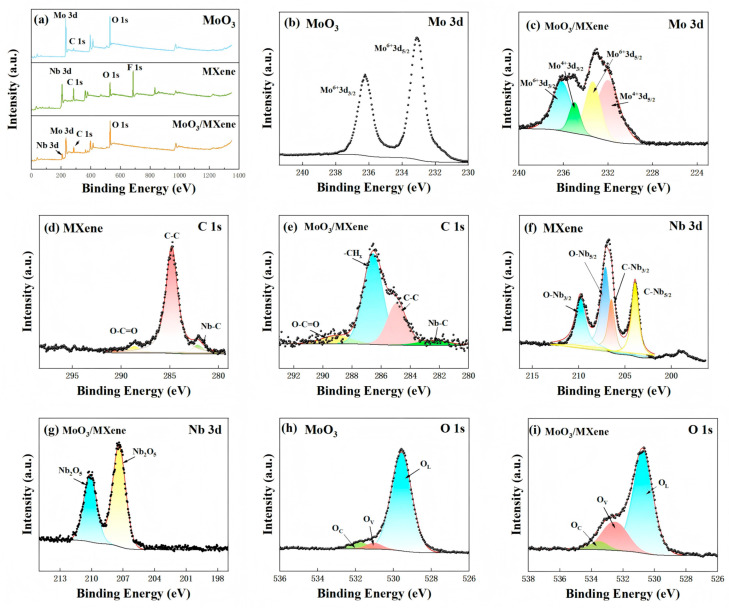
XPS spectra of pure MoO_3_ and MoO_3_/Nb_2_C composite: (**a**) survey scan; Mo 3d of MoO_3_ (**b**) and composite (**c**); C 1s of Nb_2_C (**d**) and composite (**e**); Nb 3d of Nb_2_C (**f**) and composite (**g**); O 1s of MoO_3_ (**h**) and composite (**i**).

XPS analysis confirmed the elemental composition and chemical states (Figure 4). The Mo 3d spectrum shows Mo^6+^ 3d spin-splitting doublet peaks at 233.0 and 236.3 eV. The MoO_3_ nanorod had a single valence in the spectrum. After compounding, Mo 3d shows Mo^6+^ 3d and Mo^4+^ 3d doublet peaks. The C 1s spectrum was shown in Figure 4d and e. After HF etching, the C-C peak at 284.8 eV was the main peak in Nb_2_C MXene. After the hydrothermal reaction, the peak intensity was weaker, and the -CH peak at 287 eV retained more. Pure Nb_2_C MXene shows similar Nb-C and Nb-O peak intensity in Figure 4f because Nb_2_C MXene was easily oxidized. There were only Nb-O peaks at 207 and 210 eV after compounding. The O 1s spectrum (Figure 4h) was deconvoluted into lattice oxygen (O_L_, 529.8 eV), oxygen vacancies (O_V_, 530.4 eV), and chemisorbed oxygen (O_C_, 531.6 eV). Notably, the combined proportion of O_V_ and O_C_ reached 44.18%, suggesting a relatively high density of surface active sites, which are favorable for electronic transduction during gas sensing [26].

### 3.2. Gas-Sensing Performance

**Figure 5:**
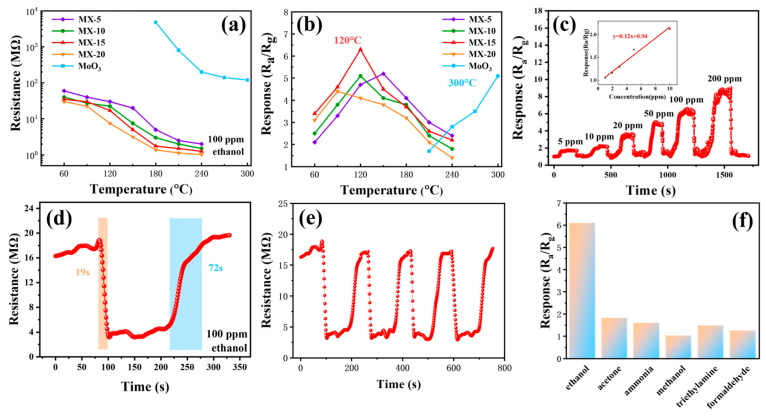
Gas-sensing performance of the sensors: (**a**) resistance–temperature curves; (**b**) response–temperature curves; (**c**) dynamic response–recovery curves; (**d**) response and recovery times; (**e**) repeatability @100 ppm; (**f**) selectivity.

The operating temperature is a critical parameter for chemiresistive sensors. As shown in Figure 5a, the baseline resistance of all sensors decreases with increasing temperature, demonstrating a typical semiconductor thermal excitation mechanism [27]. The introduction of metallic Nb_2_C constructs efficient charge transport paths, leading to significantly lower resistance in the composites compared to pure α-MoO_3_.

The response of the sensors toward 100 ppm ethanol follows a “volcano-shaped” trend with temperature (Figure 5b). Pristine α-MoO_3_ reaches its maximum response at 300 °C due to the chemical inertness of its (040) facets. In contrast, the optimal operating temperature for the MoO_3_/Nb_2_C sensors (MX-5, MX-10, MX-15, and MX-20) is reduced to approximately 120 °C. The MX-15 sensor exhibited the highest response of 6.1 with a 16 MΩ R_a_, which is comparable to those reported for many ethanol sensors (Table 1). The decreased response of MX-20 (~4.3) suggests that excessive MXene may lead to carrier transportation bypassing the interfacial depletion layer, thus reducing the sensitivity—a behavior similar to the over-loading effects reported in recent MXene-based composites [28].

**Figure 6:**
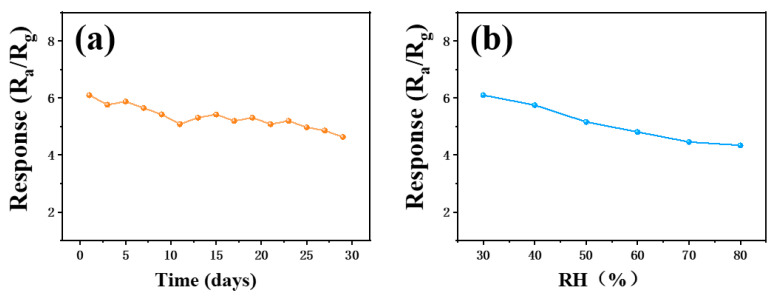
(**a**) Long-term stability over 30 days and (**b**) influence of relative humidity on the MX-15 sensor.

The MX-15 sensor demonstrated a clear concentration dependence at 120 °C, showing a linear relationship (y = 0.12x + 0.94) in the 5–200 ppm range. The sensor also exhibited rapid sensing kinetics with response and recovery times of 19 s and 72 s, respectively (Figure 5d). Furthermore, comparative tests against interfering gases (acetone, ammonia, and methanol) showed significant selectivity toward ethanol, which may be related to the enhanced interaction between ethanol hydroxyl groups and the (110) facets [25]. The long-term stability of the MX-15 sensor was evaluated over a period of 30 days. The devices were tested every 2 days and kept in a constant temperature and humidity chamber. Before testing, the devices were kept at 120 °C for over 30 min. As shown in Figure 6a, the results confirmed that response fluctuations remained within ±5%, indicating good durability. Additionally, the influence of relative humidity on the sensing performance is depicted in Figure 6b. With the rise in humidity, the R_a_ was decreased for water absorption ionization on the surface of the material, increasing carrier density. And also the water molecule occupies the active sites for gas adsorption to reduce gas response.

Humidity interference can be mitigated by a hydrophobic coating (e.g., PTFE or silane) that suppresses water adsorption while maintaining ethanol diffusion, and real-time compensation via a co-integrated humidity sensor, enabling reliable detection in variable-humidity environments like breath analysis and industrial monitoring.

### 3.3. Gas-Sensing Mechanism

**Figure 7:**
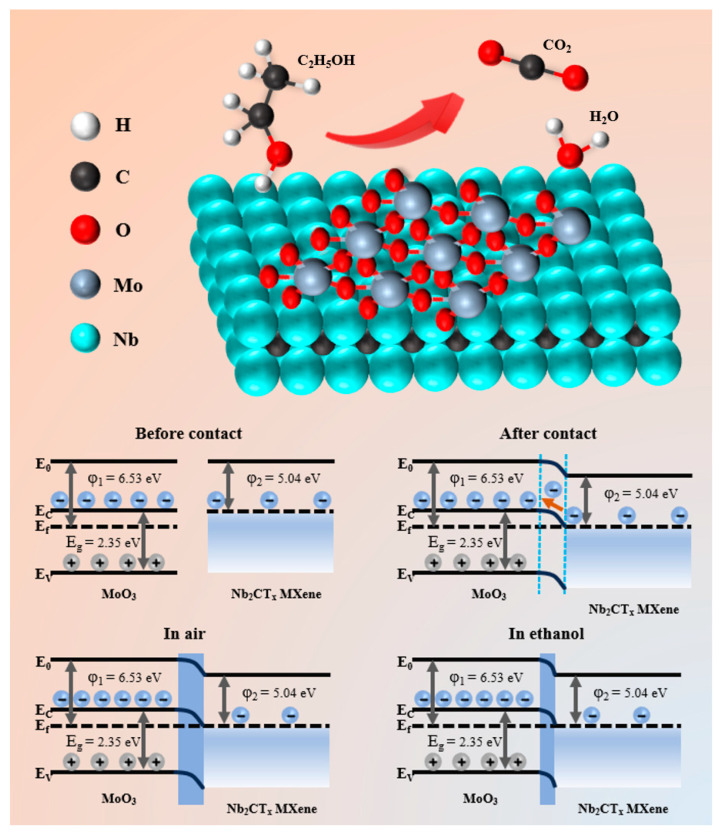
Schematic illustration of the ethanol sensing mechanism and energy band evolution for the MoO_3_/Nb_2_C MXene heterojunction.

Based on the gas-sensing performance and material characterizations, a synergistic sensing mechanism for the MoO_3_/Nb_2_C MXene composite is proposed and schematically illustrated in Figure 7. The sensing mechanism is traditionally analyzed using the surface control model, based on the interaction between chemisorbed oxygen species and target gases on the MoO_3_ surface [35]. Generally, oxygen present in ambient air is adsorbed onto the MoO_3_ surface and is converted to *O*_2_^−^, *O*^−^, and *O*^2−^ by capturing electrons near the conduction band. The surface-absorbed oxygen forms an electron depletion layer (EDL) following the reactions [36]:

(2) $$O_{2}\left(gas\right)\to O_{2}\left(ads\right)$$

(3) $$O_{2}\left(ads\right)+e^{-}\to O_{2}^{-}\left(ads\right)$$

(4) $$O_{2}^{-}\left(ads\right)+e^{-}\to 2O^{-}\left(ads\right)$$

When exposed to ethanol, the ethanol molecules react with the surface-adsorbed oxygen according to the following equation [36]:

(5) $$C_{2}H_{5}OH\left(ads\right)+6O^{-}\left(ads\right)\to 2CO_{2}\left(gas\right)+3H_{2}O\left(gas\right)+6e^{-}$$

This reaction releases electrons back into the conduction band, leading to a narrowing of the EDL and a decrease in sensor resistance.

In this work, the introduction of Nb_2_C appears to induce a facet reconfiguration of MoO_3_. Specifically, the abundant surface functional groups on Nb_2_C MXene provide numerous heterogeneous nucleation sites, which interfere with the intrinsic anisotropic growth kinetics along the [010] direction, consequently shifting the dominant planes from the chemically inert (040) to the highly active (110) and (021) facets. The DFT calculations [19,25,37] show the (040) van der Waals surface exhibits a relatively weak interaction (−0.7 eV) towards ethanol. Other facets released via complex reaction possess more negative adsorption energies and high-density coordinatively unsaturated Mo sites. This may facilitate the chemisorption of oxygen and ethanol molecules, thereby potentially lowering the reaction activation energy. Additionally, the abundant oxygen vacancies (44.18%) confirmed by XPS are generally regarded as active sites that can capture more oxygen species, enhancing the intrinsic sensitivity [38].

The integration of Nb_2_C MXene into the MoO_3_/Nb_2_C interface is anticipated to result in the formation of a nano heterojunction. The MoO_3_ work function (Φ = 6.53 eV) is higher than that of Nb_2_C (Φ = 5.04 eV) [19,39]. The Fermi level balance may drive electron injection from Nb_2_C to MoO_3_, causing band bending and the formation of an additional depletion region at the hetero-interface [40,41]. During gas interaction, both the surface EDL and the interfacial barrier are likely to be modulated, potentially amplifying the electrical signal response. Furthermore, the high conductivity of Nb_2_C provides a carrier transportation highway, which may contribute to shorter response and recovery times. The reduced response of the MX-20 sensor may be attributed to the excessive MXene loading, which causes the conductive path to bypass the interfacial depletion zones, thereby weakening the sensing sensitivity.

## 4. Conclusions

In summary, MoO_3_/Nb_2_C MXene composites were synthesized via a facile hydrothermal method. Nb_2_C MXene induces a facet reconstruction of MoO_3_ from chemically inert (040) planes to highly active (110) and (021) planes. The MX-15 sensor (15 wt% Nb_2_C) demonstrated optimal performance, with a response of 6.1 toward 100 ppm ethanol at a low operating temperature of 120 °C. The sensor also exhibited rapid response/recovery times (19 s/72 s), high selectivity, and good long-term stability. The synergistic effects, arising from the high specific surface area of the cluster structure, the exposure of highly active facets, and the efficient electron transport network established by Nb_2_C, preserve and enable the high-sensitivity and rapid-response detection of ethanol at a safe operating temperature. This work proposes a straightforward method by incorporating MXene to lower the operating temperature of the MOS sensor, demonstrating its potential for high-performance ethanol detection in industrial and traffic safety applications.

## Figures and Tables

**Table 1:** Gas-sensing performance of various sensing materials to ethanol in the literature and the present work.

Sensing Materials	Operating Temperature (°C)	Ethanol (ppm)	Response (R_a_/R_g_)	Ref.
MoO_3_/Nb_2_CT_x_ MXene	120	100	6.1	This work
MoO_3_/RGO	110	100	2.5	[29]
MoO_3_ nanoplates	400	100	13	[30]
MoO_3_ microrods	332	500	8.5	[31]
Zn doped MoO_3_ nanobelts	240	250	50	[32]
W_18_O_49_/Ti_3_C_2_T_x_ MXene	200	20	~2	[33]
Pd-SnO_2_-Graphene composites	200	100	14.8	[34]

## Data Availability

The original contributions presented in this study are included in the article. Further inquiries can be directed to the corresponding authors.
